# Body Weight Prediction from Linear Measurements of Icelandic Foals: A Machine Learning Approach

**DOI:** 10.3390/ani12101234

**Published:** 2022-05-11

**Authors:** Alicja Satoła, Jarosław Łuszczyński, Weronika Petrych, Krzysztof Satoła

**Affiliations:** 1 Department of Genetics, Animal Breeding and Ethology, Faculty of Animal Science, University of Agriculture in Krakow, al. Mickiewicza 24/28, 30-059 Krakow, Poland; jaroslaw.luszczynski@urk.edu.pl; 2 Punktur Icelandic Horses, Debowy Gaj 47, 59-600 Lwówek Śląski, Poland; punktur.weronika@gmail.com; 3 Independent Researcher, www.satola.net; krzysztof@satola.net

**Keywords:** horses, growth, models, equations, evaluation

## Abstract

**Simple Summary:**

Knowing the body weight of a growing horse is important both for horse breeders and veterinarians because this information helps to identify abnormalities of the growing process, determine adequate feeding rations or choose an appropriate drug treatment regimen. It is not always possible to measure accurately a horse’s body weight using special scales, and a visual assessment, which is the easiest method for finding out a horse’s body weight, produces heavily biased results. Simple formulas are being sought to allow making accurate estimates of body weight in horses based on their body measurements. This study relates the estimation of body weight in Icelandic foals with the use of models relying on machine learning methods. Based on their evaluation, two of the models are recommended for use in practical applications.

**Abstract:**

Knowledge of the body weight of horses permits breeders to provide appropriate feeding and care regimen and allows veterinarians to monitor the animals’ health. It is not always possible to perform an accurate measurement of the body weight of horses using horse weighbridges, and therefore, new body weight formulas based on biometric measurements are required. The objective of this study is to develop and validate models for estimating body weight in Icelandic foals using machine learning methods. The study was conducted using 312 data records of body measurements on 24 Icelandic foals (12 colts and 12 fillies) from birth to 404 days of age. The best performing model was the polynomial model that included features such as heart girth, body circumference and cannon bone circumference. The mean percentage error for this model was 4.1% based on cross-validation and 3.8% for a holdout dataset. The body weight of Icelandic foals can also be estimated using a less complex model taking a single trait defined as the square of heart girth multiplied by body circumference. The mean percentage error for this model was up to 5% both for the training and the holdout datasets. The study results suggest that machine learning methods can be considered a useful tool for designing models for the estimation of body weight in horses.

## 1. Introduction

One of the essential indicators, which could help horse breeders or owners to define an optimum feeding and horse keeping system, is the horse’s body weight [1,2,3,4]. The regular monitoring of this parameter permits not only evaluating the health and physical condition of horses, but also establishing the appropriate quantities of specific ingredients in feeding rations or the adequate drug dosage [5,6,7].

The best procedure for determining the body weight in a horse is to weigh the horse using a special horse weighbridge. However, due to high purchase prices, large size and low mobility, it is not always possible to have access to such equipment, in particular when specific procedures, which require knowing the horse’s body weight, need to be carried out outdoors [4].

One of the simplest alternatives for the determination of body weight is the method of visual assessment. The visual assessment method is the most commonly used approach among veterinarians and riders [8,9] even though it can bring bias of 20% or more [10]. A majority of horse owners also estimate the body weight of their horses based on visual assessment, which can lead to the underestimation of body weight, in particular in overweight horses [11,12,13].

The body weight of a horse can be determined with greater accuracy using special measure tapes to find out heart girth. This simple method, which is easy to use outdoors, makes it possible to estimate a horse’s body weight with an error of 5 to 10%, given the differences in body proportions as well as the differences in the ratio of bone and muscle mass to volume between horses of various types and breeds [4,10,14]. To estimate a horse’s body weight more accurately, formulas and equations were developed, which also leverage other body measurements in addition to heart girth as well as the effects of certain factors, such as type, breed, sex, age, or physiological condition [2,3,4,7,15].

The above methods for determining body weight are usually oriented towards adult horses in which somatic development has already come to an end. They appear not to be very useful, however, with respect to foals during their growth and maturation because there is a significant increase in body weight and body measurements over time, and the body proportions are considerably different as compared to adult horses [16].

According to Martin-Rosset [17], when foals are born, they present a much higher degree of development as compared to other farm animals. Given the daily weight gain of about 1–1.5 kg, foals usually double their birth weight within a period of two months. Considering the non-uniform growth speed in horses during their initial development stages, it is difficult to determine the correct body weight in foals, especially during the first six months of age, when body measurements tend to increase quite rapidly [18]. This is particularly the case with primitive horse breeds, where the development of foals, given that they are kept in an environment similar to that of wild or feral horses, might not follow the typical pattern, e.g., the pattern observed in purebred horses kept under more controlled conditions [19,20].

Bearing in mind that the existing equations designed for the estimation of body weight in growing horses are oriented towards Thoroughbred horses [16,21], it seems reasonable to develop formulas that would allow finding out the body weight in foals of primitive breeds, which include Icelandic horses. As far as foals are concerned, simple formulas based on biometric measurements, which do not require that a foal remain in the same position during the measurement, can be particularly useful. The formulas developed by Staniar et al. [16] and Rodriguez et al. [21] for the estimation of body weight of Thoroughbred foals should not be used for Icelandic foals due to the differences in the body build between the two horse breeds.

Machine learning algorithms are increasingly being used for developing models designed for use in animal science. They are widely used for predicting such diseases as subclinical ketosis or mastitis in cattle [22,23,24,25] and for the identification of heat-stressed cows [26]. As far as other animal species are concerned, machine learning methods are used, e.g., to detect parturition events in grazing sheep [27] or to predict the body weight in pigs from related feeding behavior data [28].

A machine learning approach is rather rarely used in studies concerning horses. Several attempts have been made at using machine learning methods for the recognition of facial expressions of pain in horses [29] and for the prediction of horse race results [30].

Unlike the classic statistical procedures, where the study area needs to be analyzed in depth prior to choosing variables and developing a model, machine learning methods focus on fitting data over a wide range of computationally complex models. Of relevance to the machine learning approach is the validation stage, which provides feedback on model’s performance, while the cross-validation stage helps to avoid model overfitting.

The objective of this study is to develop a model using machine learning methods, which would be useful for estimating the body weight in Icelandic foals based on biometric measurements, such as heart girth, body circumference, height at withers and cannon bone circumference. We tested linear and polynomial models that would allow breeders to easily and accurately estimate the body weight in horses without relying on a computer. We also compared these models with other computationally more complex non-linear models, which would require dedicated software for use.

## 2. Materials and Methods

### 2.1. Animals and Animal Care

The study material included a total of 24 foals (12 colts and 12 fillies) born in April and May in a stud farm of Icelandic horses in Poland. Given the pasture breeding regimen, mares foaled in the pasture where they used to remain all day and night during the pasture season. Newly born foals remained with their dams in the pasture where they had easy access to pasture herbage. The study group included foals that were born healthy and later on had no health problems that might have an adverse impact on their growth and maturation. The foals were weaned at the age of about 6 months. Once the foals were weaned, they were also kept in pastures all day and night. The foals with their dams and subsequently the weaned foals were moved once daily into the stables for several hours and given meadow hay. The measurements were taken when the foals remained in stables.

### 2.2. Morphometric Measurements

The biometric parameters and body weight of the foals (12 colts and 12 fillies) were verified at monthly intervals from birth to 404 days. From each foal 13 records were obtained for a total of 312 records (24 × 13). Height at withers was measured from the highest point of the withers vertically to the ground using a zoometric stick, while heart girth (measured around the circumference of the horse, directly behind the shoulder and over the horse’s withers), body circumference (circumference of the body across the external surfaces of the shoulder joints and points of the buttocks) and cannon bone circumference (left forelimb circumference measured in the upper third of the cannon bone) were measured using a tape measure as per the method described by Komosa and Purzyc [31]. The electronic horse weighing scale OHAUS T32XW was used to determine body weight by measurement. The error of the measuring device was 0.2 kg.

The measured variables are summarized as mean values and standard deviations according to age groups (Table 1).

### 2.3. Initial and Input Datasets

The initial dataset consisted of 312 records. Each record contained the following attributes: horse ID, sex, date of birth, date of measurements, body weight and linear body measurements, such as height at withers, heart girth, body circumference and cannon bone circumference. A variable corresponding to the foal’s age on the day of measurement was added to each record.

Parameters such as age in days, body weight and all morphometric measurements were recorded as continuous traits. Horse sex was used as a categorical variable. No records were excluded from further analysis. Finally, the input dataset contained 312 records featuring 7 attributes: body weight (dependent variable), heart girth, body circumference, height at withers, cannon bone circumference, age in days and sex.

### 2.4. Body Weight Estimation

To find most accurate and simple models that could be used by horse breeders to predict the body weight of horses, first linear models, then polynomial models, and finally non-linear models were created using different machine learning (ML) algorithms, various input feature sets, and model hyper-parameter tuning methods.

The analysis and modelling were performed using Python version 3.8 and the pandas (1.1.2), numpy (1.19.2), scipy (1.5.2), scikit-learn (0.23.2), lightgbm (3.0.0) and xgboost (1.2.0) libraries.

### 2.5. Feature Selection and Engineering

Before modelling, 62 sets of input features were created using heart girth, body circumference, height at withers, cannon bone circumference, age in days and sex. Five sets included only one feature (heart girth, body circumference, height at withers, cannon bone circumference, or age in days). There were 15 sets containing 2 features, 20 sets—three features, 15 sets—four features, six sets—five features and one set including all the six features.

In addition, a new complex feature was created based on the following formula: the square of heart girth multiplied by body circumference (HG^2^ × BC). In total, including the new complex feature, there were 63 sets of input features used for modelling.

### 2.6. Training and Testing Datasets

The input dataset was split into training (75% of observations, 234 records) and testing (25% of observations, 78 records) subsets. To eliminate any potential data leakage, the test subset was created by choosing all observations for six randomly chosen horses: three colts and three fillies. The same training and testing subsets were used for training and testing of all the models.

### 2.7. Modelling

A total of 13 linear regression scikit-learn methods were used for modelling: DummyRegressor (always returns the mean value), LinearRegression, Ridge, Lasso, Lars, LassoLars, ElasticNet, HuberRegressor, BayesianRidge, TweedieRegressor, RANSACRegressor, TheilSenRegressor and PLSRegression. There were 819 trained linear regression models (each of the 13 methods were used for all the 63 input feature sets).

Polynomial models were trained using scikit-learn pipelines with the second degree of PolynomialFeatures and LinearRegression steps. Only 31 sets having the following features were used for training polynomial models: heart girth, body circumference, height at withers, cannon bone circumference and age in days (5 sets with 1 feature, 10 sets with 2 features, 10 sets with 3 features, 5 sets with 4 features and 1 set with 5 features). A total of 31 polynomial models were trained using the LinearRegression method.

There were nine non-linear regression methods used for modelling: seven from the scikit-learn library (SVR, KNeighborsRegressor, DecisionTreeRegressor, RandomForestRegressor, AdaBoostRegressor, BaggingRegressor, ExtraTreesRegressor) and two (LGBMRegressor, XGBRegressor) from the lightgbm and xgboost libraries. There were 567 trained non-linear regression models (each of the 9 methods was used with all the 63 input feature sets).

Each model was fine-tuned multiple times using different sets of hyper-parameters and the scikit-learn GridSearchCV method. Models trained using the training dataset were evaluated based on 10-fold cross-validation (CV) repeated 10 times with the mean coefficient of determination, mean absolute error, root mean square error, mean percentage error (defined in the next paragraph) and their standard deviations as model performance metrics. Next, the best performing models were fitted to the entire training dataset for making predictions at a later stage (using unseen data represented by the testing dataset). The final ranking of the models was carried out separately for each of the four groups of models (linear, polynomial, linear with the new complex input feature and non-linear) based on the mean percentage error from cross-validation (with lower values corresponding to the best performing models). At the end, one best performing model was selected from each group.

The testing (holdout) dataset was later used to compare the best performing models using unseen data.

### 2.8. Evaluation Metrics

The best performing models were chosen using four diagnostic criteria: coefficient of determination (R^2^), root-mean-square error (RMSE), mean absolute error (MAE) and mean percentage error (MPE), defined as follows:

(1) $$R^{2}=\frac{\sum {\left({\hat{y}}_{i}-\bar{y}\right)}^{2}}{\sum {\left(y_{i}-\bar{y}\right)}^{2}}$$

(2) $$\mathrm{RMSE}=\sqrt{\frac{\sum e_{i}^{2}}{n}}$$

(3) $$\mathrm{MAE}=\frac{\sum \left|e_{i}\right|}{n}$$

(4) $$\mathrm{MPE}=\frac{\sum \frac{\left|e_{i}\right|}{y_{i}}\cdot 100}{n}$$

Errors ($e_{i}$) were calculated as a difference between the measured ($y_{i}$) and the predicted (${\hat{y}}_{i}$) body weight of a horse, where $\bar{y}$ was a mean value of the measured body weights $y_{i}$.

## 3. Results

Table 2 shows the mean values of the evaluation metrics (RMSE, MAE, R^2^ and MPE), including the related standard deviations, from the cross-validation for the best performing linear models featuring only one of the independent variables: heart girth, height at withers, body circumference, cannon bone circumference or age in days. The values of the individual metrics were also calculated for the test dataset. The lowest R^2^ (0.77) from the cross-validation was obtained for the model using age as the only independent variable and for the model taking cannon bone circumference as the only predictor. The R^2^ for the models referred to above were also lowest (0.78) for the test dataset (Table 2). The MPE from the cross-validation for the model using only age in days was 16.4%, which was the highest error among those obtained for models taking a single independent variable as input. This demonstrates that age is a weak predictor of body weight in Icelandic horses during their first year following birth. Body circumference and heart girth were found to be the best predictors of body weight in Icelandic horses. For models based on these features, R^2^ from the cross-validation was 0.94 and 0.92, respectively, while the respective values for the testing dataset were 0.93 and 0.94 (Table 2). For the model using body circumference as the only independent variable, the MPE from the cross-validation was 7.3%. A similar percentage error (7.6%) was obtained for the model featuring only heart girth. The other metrics (RMSE and MAE) were also lower than those for the model using age in days as the only independent variable (Table 2).

Next, we tested linear models using several input variables, polynomial models (quadratic polynomials), other non-linear models and linear models using the new complex feature given as the square of heart girth multiplied by body circumference. Table 3 shows the mean values of metrics (and corresponding standard deviations) for the best performing linear, polynomial and non-linear models, and the best performing linear model using the new complex feature, as well as the values of these metrics for the testing dataset. Below are specified the estimated regression equations for the best performing linear model (M1), polynomial model (M2) and linear model featuring one independent variable given as the square of heart girth multiplied by body circumference (M3):

(M1) BW=1.032×HG+0.503×BC+0.084×AGE−119.514

BW—body weight (kg)

HG—heart girth (cm)

BC—body circumference (cm)

AGE—age in days

$$(M2)\;\mathrm{BW}=0.276\times \mathrm{HG}-0.832\times \mathrm{BC}-10.170\times \mathrm{CC}+0.011\times \mathrm{HG}\times \mathrm{CC}+0.054\times \mathrm{BC}\times \;\mathrm{CC}-0.004\times \mathrm{HG}\times \mathrm{BC}+0.007\times {\left(\mathrm{HG}\right)}^{2}+0.003\times {\left(\mathrm{BC}\right)}^{2}-0.030\times {\left(\mathrm{CC}\right)}^{2}+102.628$$

BW—body weight (kg)

HG—heart girth (cm)

BC—body circumference (cm)

CC—cannon bone circumference (cm)

$$(M3)\;\mathrm{BW}=\frac{{\left(\mathrm{HG}\right)}^{2}\times \mathrm{BC}}{26583.655}+9.453$$

BW—body weight (kg)

HG—heart girth (cm)

BC—body circumference (cm)

The parameters of the linear model M1 were estimated using the Ridge regression method, the parameters of the polynomial model M2 using the LinearRegression method, and the parameters of the model M3 using the Lars regression method.

**Table 3:** Root-mean-square error (RMSE), mean absolute error (MAE), coefficient of determination (R^2^) and mean percentage error (MPE) from the cross-validation using the training and testing datasets for four best performing models for predicting body weight of Icelandic foals: linear model featuring three traits (M1), quadratic polynomial (M2), linear model featuring a complex trait (M3) and a non-linear model based on the ExtraTreesRegressor algorithm (M4).

Model Type	Features	Training (Mean ± SD)				Testing			
		RMSE	MAE	R^2^	MPE	RMSE	MAE	R^2^	MPE
M1	HG, BC, AGE	9.10 ± 1.32	7.20 ± 1.15	0.95 ± 0.02	5.4 ± 0.8	8.98	6.90	0.96	4.8
M2	HG, BC, CC	7.12 ± 1.17	5.54 ± 0.92	0.97 ± 0.01	4.1 ± 0.7	6.94	5.20	0.98	3.8
M3	(HG)^2^ × BC	8.24 ± 1.31	6.46 ± 1.02	0.96 ± 0.02	4.9 ± 0.7	8.49	6.49	0.97	4.7
M4	HG, BC, HW, CC, AGE, SEX	6.25 ± 1.37	4.68 ± 0.76	0.98 ± 0.01	3.6 ± 0.6	7.67	5.82	0.97	4.6

Model M1: BW=1.032×HG+0.503×BC+0.084×AGE−119.514; Model M2: $\mathrm{BW}=0.276\times \mathrm{HG}-0.832\times \mathrm{BC}-10.170\times \mathrm{CC}+0.011\times \mathrm{HG}\times \mathrm{CC}+0.054\times \mathrm{BC}\times \mathrm{CC}-0.004\times \mathrm{HG}\times \mathrm{BC}+0.007\times \;{\left(\mathrm{HG}\right)}^{2}+0.003\times {\left(\mathrm{BC}\right)}^{2}-0.030\times {\left(\mathrm{CC}\right)}^{2}+102.628$; Model M3: $\mathrm{BW}=\frac{{\left(\mathrm{HG}\right)}^{2}\times \mathrm{BC}}{26583.655}+9.453$; HG, heart girth; BC, body circumference; CC, cannon bone circumference; HW, height at withers; BW, body weight.

Table 4 shows the specification of the best performing non-linear model M4 based on the ExtraTreesRegressor algorithm.

The highest RMSE and MAE from the cross-validation were obtained for the linear model M1 (9.10 and 7.20, respectively); slightly lower values were obtained for the polynomial model M2 (7.12 and 5.54, respectively), while the non-linear model M4 was found to be the best performing model (RMSE = 6.25 and MAE = 4.68), considering the values of the metrics referred to above (Table 3). The non-linear model was also characterized by the highest mean R^2^ (0.98) and the lowest MPE from the cross-validation (3.6%). The external validation based on the testing dataset for the non-linear model resulted in higher errors (RMSE, MAE and MPE) than those arising from the cross-validation, while the values of the metrics being analyzed were similar both for the training and the testing datasets for the polynomial model M2.

Table 3 also shows the values of the individual metrics for the linear model M3 featuring the new complex feature defined as the square of heart girth multiplied by body circumference. The mean values of errors from the cross-validation (RMSE = 8.24 and MAE = 6.46) were lower than those obtained for other linear models featuring a single independent variable (Table 2). The MPE was 4.9% and the mean R^2^ for this model was 0.96. Similar values of the metrics being analyzed were also obtained for the testing dataset, which might indicate that this model is a good prediction model.

**Figure 1:**
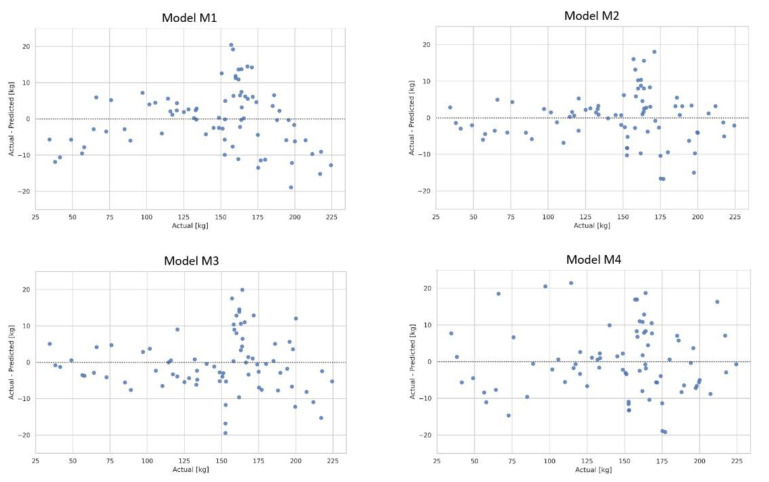
The residual plots of the body weight target feature on testing data fitted to four different models: linear model featuring three traits (**M1**), quadratic polynomial (**M2**), linear model featuring a complex trait (**M3**) and a non-linear model based on the ExtraTreesRegressor algorithm (**M4**).

Graphs of the distribution of errors for the testing dataset (Figure 1) were produced for the four models referred to above (M1, M2, M3 and M4). The smallest differences between the actual and the estimated body weight were observed for the polynomial model M2, and they were no greater than 7.8 kg for horses weighing up to 150 kg. For horses weighing more than 150 kg, the maximum difference between the actual and the estimated body weight was 18.4 kg.

## 4. Discussion

Age in days is a weak predictor of body weight in horses [3]. As demonstrated in our study, this also refers to Icelandic horses (R^2^ = 0.77) (Table 2). The first year of age in horses is characterized by rapid growth and body weight gains [16], and therefore a linear function is not the best choice for describing this phenomenon. Body circumference and heart girth were found to be much better predictors of body weight in Icelandic foals (R^2^ = 0.94 and R^2^ = 0.92 from the cross-validation) (Table 2). The formulas proposed by Rodriguez et al. [21] for the estimation of the body weight of Thoroughbred foals also featured only one independent variable, namely, heart girth. The authors presented three models adapted to data, linear, exponential and linear, taking the input feature given as the cube of heart girth. The highest R^2^ was obtained for the latter of the models referred to above (0.98), while slightly lower values were obtained for the exponential model (0.97) and the linear model (0.96). Rodriguez et al. [21] highlighted that their body weight estimation formula for Thoroughbred foals featured no such traits as body length or height at withers due to the difficulty in performing the respective body measurements. A strong correlation between a horse’s heart girth and body weight was the reason why tape measure became very popular and are widely used for estimating body weight in horses.

Taking as an example the study of Staniar et al. [16], we devised a new complex feature, which is the square of heart girth multiplied by body circumference (HG^2^ × BC), and we estimated coefficients of a linear regression model based solely on this one feature. The formulas published by Staniar et al. [16] assume that the body weight of a horse can be estimated from cylindrical body (barrel) volume multiplied by a density factor, where body volume can be estimated from heart girth and body length. A similar approach to the estimation of body weight in horses, such as the one presented by Staniar et al. [16], was used by Carroll and Huntington [32]. In our study, instead of body length (which was not measured in the Icelandic horses included in the study group), we used body circumference, which is a multiple of body length. The measurement of body circumference in foals is easier as compared to the measurement of body length. To measure correctly the body length of a horse, the animal should remain in a specific position (with all the legs on the ground), which can be difficult to achieve in foals because they are very easily disturbed. The linear model M3 (Table 3), using a single feature given as the square of heart girth and body circumference, achieved in the cross-validation a higher R^2^ (0.96) and lower MPE (4.9) as compared to the other linear models featuring a single predictor (Table 2). Similar values of the metrics being analyzed for the model M3 were also obtained from the test dataset representing unseen data (Table 3). This relatively simple model could be used for estimating body weight in Icelandic foals.

Some simple formulas for the estimation of a horse’s body weight were pointed out by Jensen et al. [33]. They compared the accuracy of the different formulas designed for the estimation of body weight in adult Icelandic horses and adult warmbloods (4 years of age or older). The authors found that some simple formulas featuring only heart girth or heart girth combined with body length can be used for estimating body weight in Icelandic and warmblood horses just as well as more complex formulas using such traits as height at withers or neck circumference. To take an example, using the formula proposed by Martinson et al. [2], which included traits such as height at withers, neck circumference, heart girth and body length, Jensen et al. [33] obtained for Icelandic horses an RMSE of 19.6 kg, while the simpler formula proposed by Carroll and Huntington [32], which included only heart girth and body length, resulted in a slightly greater RMSE of 21.1 kg.

In our study, aside from simple linear models featuring one independent variable, we also tested linear models taking several input variables as well as polynomial models and other non-linear models, and we evaluated the predictive potential of these study models. The best performing linear model M1 for the estimation of body weight in foals featured three variables: heart girth, body circumference and age in days (Table 3). This model was worse performing (based on the mean values of R^2^, RMSE, MAE and MPE from the cross-validation) than the linear model M3 that used only the complex feature given as the square of heart girth multiplied by body circumference. Linear models are rarely used for making estimates of horses’ body weight. Catalano et al. [34] used a linear model to estimate an ideal body weight of draft horses and warmbloods, taking into account such traits as height at withers and body length. However, they obtained quite a low R^2^ (0.90) for this model.

The polynomial model M2 (quadratic polynomial) featuring such input variables as heart girth, body circumference and cannon bone circumference, and the non-linear model M4 based on the ExtraTreesRegressor algorithm and featuring such input variables as heart girth, body circumference, height at withers, cannon bone circumference, age in days and sex were found to be better performing models as compared to the linear model M1 (Table 3). The polynomial model M2 is recommended for use in practical applications related to the estimation of body weight in Icelandic foals up to 13 months of age (Table 3). The polynomial model, even though computationally complex, can be used by horse owners and breeders or veterinarians for the estimation of body weight in Icelandic foals without the need to rely on a computer, while the use of the non-linear model M4 would require using specifically dedicated software. The use of the model M3 using a single input feature given as the square of heart girth multiplied by body circumference should also be taken into consideration. This model is characterized by lower accuracy as compared to the polynomial model M2; however, it enables the easier and less time-consuming estimation of body weight in horses. Both models should be used only for Icelandic foals (colts and fillies) because they were fitted and validated for this breed.

## 5. Conclusions

The body weight in Icelandic foals aged 13 months or less can be estimated using a simple formula using a single feature given as the square of heart girth multiplied by body circumference, for which the mean percentage error was about 5%, as well as using a more complex polynomial model (a quadratic polynomial) taking input features such as heart girth, body circumference and cannon bone circumference, for which the mean percentage error was about 4%. Both formulas can be employed without the need to use any dedicated software. Additional research studies are needed to analyze if the proposed models make it possible to estimate precisely the body weight in Icelandic foals. The expansion of the database containing results of body weight and biometric measurements in Icelandic foals may involve the need to re-estimate the coefficients and re-evaluate the models.

## Figures and Tables

**Table 1:** The growth variables defined for Icelandic foals, summarized as mean values and standard deviations according to age groups.

Age Group (Days from Birth)	Number of Observations (Number of Foals)	Body Weight [kg]	Heart Girth [cm]	Body Circumference [cm]	Height at Withers [cm]	Cannon Bone Circumference [cm]
1–30	32 (24)	49.9 ± 14.3	80.9 ± 7.3	166.9 ± 15.7	88.5 ± 5.0	12.0 ± 0.6
31–60	16 (16)	72.4 ± 5.2	92.3 ± 3.4	196.4 ± 6.5	97.7 ± 3.3	12.4 ± 0.5
61–90	27 (23)	108.3 ± 14.4	106.3 ± 6.0	228.0 ± 10.8	103.1 ± 2.5	13.3 ± 0.6
91–120	20 (20)	123.9 ± 14.5	111.0 ± 4.9	240.7 ± 8.3	106.3 ± 2.4	13.4 ± 0.6
121–150	26 (24)	136.6 ± 15.2	115.5 ± 5.3	248.9 ± 7.2	107.5 ± 3.3	14.2 ± 0.5
151–180	22 (22)	150.4 ± 15.3	121.3 ± 4.7	254.0 ± 6.9	110.3 ± 2.7	14.3 ± 0.5
181–210	23 (23)	164.9 ± 14.1	123.8 ± 4.8	262.6 ± 9.7	112.4 ± 2.2	14.6 ± 0.5
211–240	22 (22)	163.9 ± 12.7	124.7 ± 4.4	262.9 ± 9.4	113.4 ± 2.2	14.6 ± 0.5
241–270	23 (23)	165.0 ± 14.1	126.7 ± 5.6	265.0 ± 9.0	114.7 ± 2.2	14.6 ± 0.4
271–300	27 (24)	165.1 ± 15.2	125.4 ± 5.1	268.5 ± 9.9	115.9 ± 3.0	14.5 ± 0.4
301–330	21 (21)	162.9 ± 13.9	124.4 ± 4.5	268.7 ± 9.5	117.1 ± 3.0	14.8 ± 0.5
331–360	24 (24)	179.0 ± 14.9	127.3 ± 4.0	278.4 ± 8.3	118.7 ± 2.8	15.2 ± 0.5
361–404	29 (24)	201.8 ± 16.9	131.8 ± 4.5	285.9 ± 10.0	120.1 ± 3.0	15.8 ± 0.7

**Table 2:** Root-mean-square error (RMSE), mean absolute error (MAE), coefficient of determination (R^2^) and mean percentage error (MPE) from the cross-validation using the training and testing datasets for linear models featuring one independent variable for the prediction of body weight in Icelandic foals.

Feature	Training (Mean ± SD)				Testing			
	RMSE	MAE	R^2^	MPE	RMSE	MAE	R^2^	MPE
Heart girth [cm]	11.94 ± 2.12	9.60 ± 1.71	0.92 ± 0.03	7.6 ± 1.5	11.22	8.40	0.94	6.0
Body circumference [cm]	10.62 ± 1.68	8.60 ± 1.18	0.94 ± 0.03	7.3 ± 1.3	11.91	9.56	0.93	7.6
Height at withers [cm]	13.94 ± 1.76	11.28 ± 1.56	0.89 ± 0.04	9.5 ± 2.1	11.62	9.27	0.93	7.1
Cannon bone circumference [cm]	20.09 ± 2.61	16.43 ± 2.40	0.77 ± 0.09	14.9 ± 3.4	21.44	16.93	0.78	14.8
Age in days	20.50 ± 2.46	16.98 ± 2.48	0.77 ± 0.10	16.4 ± 4.3	21.15	17.17	0.78	15.2

**Table 4:** Hyper-parameters used for building the best performing non-linear model (M4).

Model Name	Hyperparameters
ExtraTreesRegressor (M4)	bootstrap = False, ccp_alpha = 0.0, criterion = ‘mse’, max_depth = None, max_features = ‘auto’, max_leaf_nodes = None, max_samples = None, min_impurity_decrease = 0.0, min_impurity_split = None, min_samples_leaf = 1, min_samples_split = 2, min_weight_fraction_leaf = 0.0, n_estimators = 400, n_jobs = −1, oob_score = False, random_state = 42, verbose = 0, warm_start = True

## Data Availability

The data are publicly unavailable due to data confidentiality.
